# Incidence and risk factors for chronic kidney disease in individuals with type 1 diabetes: A population‐based study in Salford, Manchester

**DOI:** 10.1111/dme.70175

**Published:** 2025-11-20

**Authors:** Hellena Hailu Habte‐Asres, Mike Stedman, Angus Forbes, Janaka Karalliedde, David C. Wheeler, Adrian H. Heald

**Affiliations:** ^1^ Florence Nightingale Faculty of Nursing, Midwifery and Palliative Care, King's College London London UK; ^2^ Royal Free London NHS Foundation Trust London UK; ^3^ Res Consortium Andover UK; ^4^ School of Cardiovascular Medicine & Sciences, King's College London London UK; ^5^ UCL Department of Nephrology, University College London, Royal Free Campus London UK; ^6^ The School of Medicine and Manchester Academic Health Sciences Centre, Manchester University Manchester UK; ^7^ Diabetes and Endocrinology, Salford Royal Hospital Salford UK

**Keywords:** chronic kidney disease, CKD, diabetic kidney disease, DKD, T1D, type 1 diabetes

## Abstract

**Aims:**

Chronic kidney disease (CKD) is a serious and common complication of type 1 diabetes (T1D). Despite improvements in diabetes management, the prevalence of CKD in people with T1D remains high. However, large‐scale, real‐world data from the UK are limited. This study aimed to provide contemporary estimates of CKD incidence and its associated risk factors in a population with T1D in Salford, Greater Manchester.

**Methods:**

We conducted a retrospective cohort study using anonymised electronic health records from the Salford Integrated Record Research Database between 2010 and 2023. Adults with T1D were assessed for CKD, defined as an estimated glomerular filtration rate (eGFR) <60 mL/min/1.73 m^2^ and/or albuminuria (urine albumin‐to‐creatinine ratio ≥3 mg/mmol), confirmed by two readings at least 90 days apart. Competing risk regression models (accounting for death as a competing event) were used to identify associations with demographic and clinical risk factors, including age, sex, ethnicity, deprivation, smoking, body mass index (BMI), HbA1c and use of ACE inhibitors or ARBs.

**Results:**

Among 1106 adults with T1D, 38.5% developed reduced eGFR, 47.2% had albuminuria, and 23.8% met both criteria for CKD. Older age was strongly associated with reduced eGFR: compared to those aged 18–28 years, the sub‐distribution hazard ratio (sHR) was 3.1 (95% CI: 2.0–5.0) for ages 42–54 and 8.6 (95% CI: 5.4–13.9) for ≥55 years. Other significant predictors included missing ethnicity data (sHR 1.5), higher BMI (sHR 1.1 per kg/m^2^), higher HbA1c (sHR 1.1 per mmol/mol) and not being prescribed an ACE inhibitor or ARB (sHR 1.4).

For albuminuria, increased risk was associated with female sex (sHR 1.3), missing ethnicity (sHR 1.4), higher HbA1c (sHR 1.1), current smoking (sHR 1.5), living in the second most deprived quintile (sHR 1.9) and being prescribed ACE inhibitors or ARBs (sHR 2.1).

**Conclusion:**

This large UK cohort study highlights a high burden of CKD among adults with T1D. Key risk factors included older age, higher BMI, poor glycaemic control, smoking and deprivation. Although ACE inhibitors and ARBs were commonly prescribed, the uptake of newer reno‐protective treatments was low. These findings point to a need for earlier detection, targeted interventions and broader implementation of effective therapies to reduce kidney disease burden in this population.

**What‘s new?**

- Higher CKD Prevalence: 38.5% of participants with T1D exhibited reduced kidney function & 47.2% had albuminuria, indicating a substantial burden of CKD in this population.
- Key Risk Factors: significant predictors of CKD include older age, higher BMI, suboptimal glycaemic levels and smoking.
- Underutilisation of Renoprotective Treatment: Despite common prescription of ACE inhibitors & ARBs, the study indicates low uptake of newer renoprotective therapies like SGLT2i & GLP1RA among individuals with T1D.

## 1 INTRODUCTION

Type 1 diabetes (T1D) is a chronic condition affecting over 400,000 people in the UK and is associated with an increased risk of microvascular complications. Chronic kidney disease (CKD) is one of the most prevalent among these, affecting approximately 30%–40% of individuals with T1D.^1^, ^2^ People with both T1D and CKD face a significantly higher risk of progressing to kidney failure and are more likely to experience cardiovascular events.^1^ For instance, data from Finland show that 36% of individuals with severe albuminuria progress to kidney failure within 15 years of its onset.^3^

Despite advances in managing hyperglycaemia and hypertension, CKD remains a serious and common complication of diabetes. Previous studies assessing CKD prevalence in T1D have been limited by reliance on administrative codes, incomplete laboratory data particularly regarding albuminuria and small sample sizes, restricting the generalisability of their findings.

While large‐scale research on CKD prevalence in T1D exists from the US^4^, ^5^ comparable population‐based data from the UK are lacking. This gap hinders the development of evidence‐based strategies to address the growing burden of CKD in people with T1D in the UK, where ethnic and sociocultural factors are variant from those in other countries. There is a recognised disparity in access to cardiorenal protective therapies for people with T1D. Although recent pharmacological advances have improved outcomes for those with type 2 diabetes (T2D), individuals with T1D have often been excluded from clinical trials of these newer treatments, limiting their access to such benefits. This highlights the need for real‐world evidence to identify individuals with T1D who may benefit from cardiorenal protective therapies.

This paper presents an analysis of a large dataset from Northwest England to estimate the incidence and characteristics of CKD in people with T1D, with the aim of informing clinical needs and identifying opportunities for interventions to improve kidney health outcomes in this population.

## 2 METHODS

We conducted a population‐based, retrospective cohort study of all adults diagnosed with T1D and living in Salford, Manchester. This cohort was initially identified in 2010 and subsequently followed prospectively until 2023. The Salford Integrated Record Research Database is a comprehensive, anonymised electronic health record database established in 2009. It captures detailed patient‐level clinical information, including consultations, test results, prescribed therapies, diagnostic codes, outpatient attendances and referrals, using the medical index codes (Read Codes or SNOMED codes) that are in the UK. Practice‐level IMD scores were used to estimate deprivation in our analysis.

The study protocol was approved by Salford Royal Foundation Trust and local general practices, with ethical approval obtained from the West of Scotland REC 4 in February 2024 (Ref: 23/WS/1075; IRAS: 324632).

### 2.1 Study population

The study population consisted of adults (aged ≥18 years) with a diagnostic code for T1D recorded between 1 January 2010 and 31 December 2023. Diabetes status was confirmed using Read diagnostic codes or SNOMED codes, applying a previously validated algorithm.^6^ T1D was further verified by checking HbA1c ≥48 mmol/mol and/or a continuous prescription of insulin, along with the absence of a Read code or SNOMED code for T2D or other forms of diabetes.

### 2.2 Eligibility criteria

Individuals were included in the study if they met all the following criteria:

- Aged ≥18 years during the study period
- Diagnosed with T1D based on validated clinical codes or an HbA1c level ≥48 mmol/mol, along with continuous insulin use.
- Registered with a Salford GP or a provider contributing to the Salford Integrated Record for ≥1 year between 2010 and 2023
- Had at least two recorded estimated glomerular filtration rate (eGFR) or ACR measurements, at least 90 days apart
- Alive on 1 January 2010

Exclusion criteria were as follows:

- Individuals aged under 18 years
- Individuals with a diagnosis of gestational diabetes or T2D
- Individuals with a history of kidney transplantation or renal replacement therapy, as identified using Read Codes or SNOMED codes

For each patient, eGFR was calculated from serum creatinine values using the CKD Epidemiology Collaboration (CKD‐EPI 2021) equation. For records prior to 2014, serum creatinine values were multiplied by 0.95 to account for the lack of calibration in creatinine reporting before that year.

CKD is defined according to international guidelines as either an eGFR of less than 60 mL/min/1.73 m^2^ sustained for at least 3 months or the presence of other markers of kidney damage, specifically a urine albumin‐to‐creatinine ratio (uACR) of 3 mg/mmol or higher.

### 2.3 Outcomes

The primary outcome of this population‐based study was the prevalence of CKD in individuals with T1D, defined as an eGFR of <60 mL/min/1.73 m^2^ and/or the presence of persistent albuminuria (uACR ≥3.0 mg/mmol), in accordance with international kidney disease: Improving Global Outcomes (KDIGO)^7^ guidelines. We reported outcomes separately for individuals with persistent eGFR <60 mL/min and those with persistent uACR ≥3 mg/mmol, either with eGFR <60 mL/min or preserved eGFR ≥60 mL/min.

### 2.4 Covariates

The analysis was adjusted for the following covariates: age, sex, ethnicity, Index of Multiple Deprivation (IMD), mean baseline body mass index (BMI), baseline mean HbA1c and use of ACE inhibitors or ARBs at baseline. Age was categorised into quartiles. Self‐reported ethnicity, identified using Read codes and based on the 2001 and 2011 UK census classifications, was used to derive a four‐category variable (White, Mixed heritage, ethnic minority). Patients without recorded ethnicity were assigned to the ‘Missing’ category. Smoking status was categorised as never smoked, ex‐smoker or current smoker.

Practice‐level IMD was used as the measure of deprivation, categorised from 1 (least deprived) to 5 (most deprived). Data were also retrieved on the use of ACE inhibitors or ARBs, GLP‐1 receptor agonists (GLP‐1 RA) and SGLT2 inhibitors (SGLT2i). Outpatient attendances and referral data were derived from Read or SNOMED codes from clinical specialties such as endocrinology, cardiology or renal medicine, and were categorised accordingly in the analysis.

### 2.5 Statistical analysis

Descriptive statistics were used to summarise baseline demographic and clinical characteristics. Continuous variables were presented as means and standard deviations (SD), while categorical variables were summarised using frequencies and percentages.

Competing risk regression models using the Fine and Gray method were employed to examine factors associated with the onset of CKD, defined separately as:

1. an eGFR <60 mL/min/1.73 m^2^; and
2. a uACR ≥3 mg/mmol.

These models accounted for death as a competing event. Sub‐distribution hazard ratios (sHRs) and corresponding 95% confidence intervals (CIs) were reported. Covariates included in the models were selected based on clinical relevance, availability of variables and existing literature and comprised age, sex, ethnicity, IMD, smoking status, BMI, mean HbA1c and use of ACE or ARB.

The percentage of patients with missing data in variables other than ethnicity was very low; therefore, we undertook a complete case analysis. Individuals with missing ethnicity data were retained in the analysis by assigning them to a ‘Missing’ ethnicity category. All statistical analyses were conducted using Stata version 19 (Stata Corp, College Station, TX, USA).

## 3 RESULTS

**FIGURE 1:**
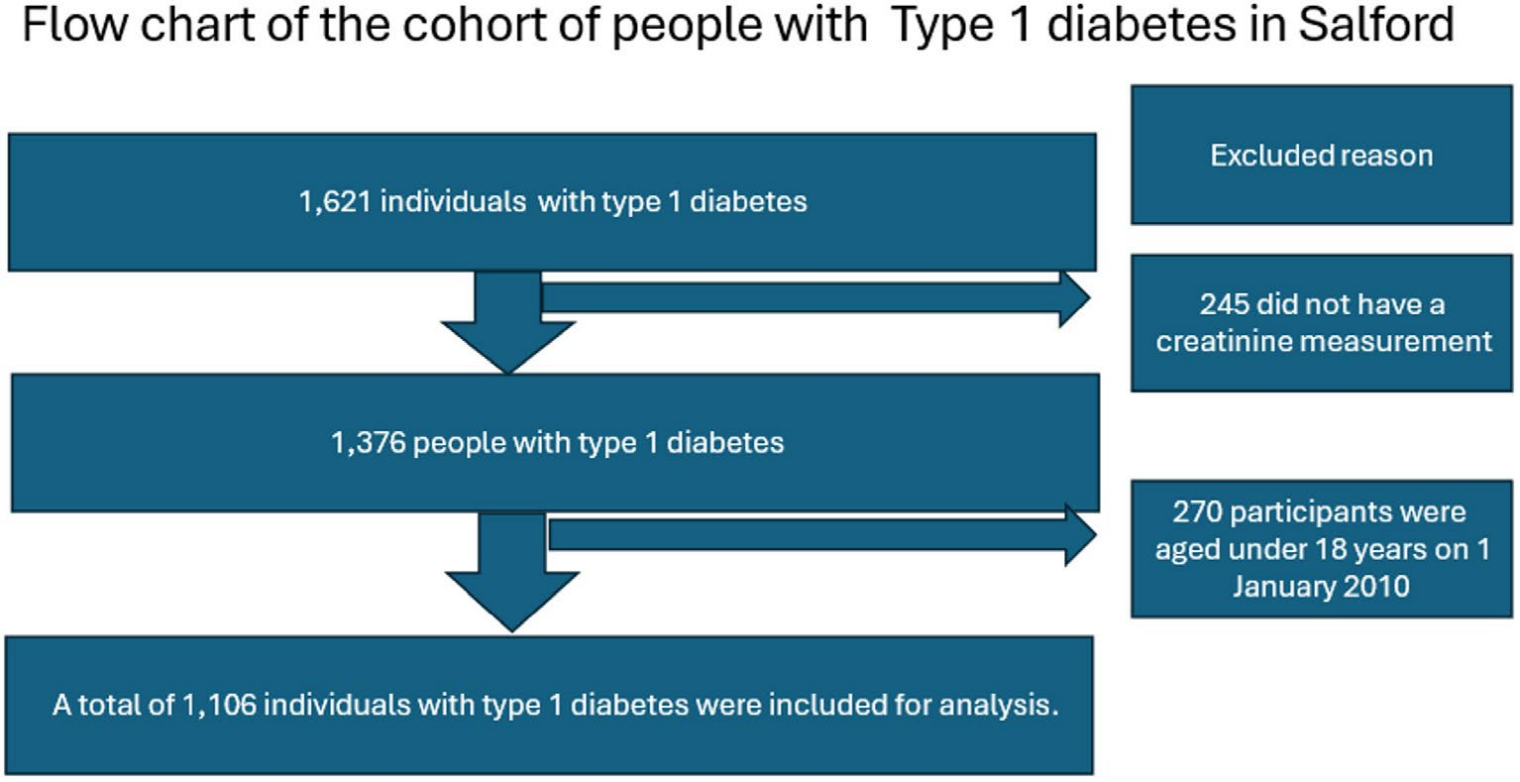
Flow chart of the cohort.

The study included 1106 participants with T1D. Details of exclusions from the source dataset are summarised in Figure 1.

Of the 1106 participants, 426 (38.5%) developed CKD over a mean follow‐up of 7.4 years (SD ± 4.5). During this period, 237 participants died. Those with CKD were, on average, older, with a mean age of 56.7 years compared with 36.1 years among those without CKD, and a slightly higher proportion of females had CKD (45.5% vs. 39.0%). The ethnic distribution was broadly similar; however, a smaller proportion of participants from ethnic minority backgrounds had CKD (2.8% vs. 7.7%), and missing ethnicity data were more common in the CKD group (13.4%). CKD presents earlier in ethnic minority populations (mean age 29.7 years) than in white individuals (35.4 years). Nearly half of all participants lived in the most deprived quintile of the IMD, with a slightly higher proportion among those with CKD (50.7% vs. 48.2%). Differences emerged in smoking status: participants with CKD were more likely to be ex‐smokers (43.7% vs. 27.9%), whereas current smoking was more prevalent among those without CKD (33.8% vs. 24.4%).

Mean BMI was marginally higher in the CKD group (28.3 vs. 27.0 kg/m^2^). As expected, the mean baseline eGFR was markedly lower in the CKD group (33.5 vs. 89.1 mL/min/1.73 m^2^), while the uACR was considerably higher (15.9 vs. 0.5 mg/mmol); however, HbA1c levels were comparable between groups. The use of ACE inhibitors or ARBs was substantially greater among participants with CKD (73.2% vs. 40.2%), and SGLT2i use was also higher (15.5% vs. 11.3%), while GLP‐1 receptor agonist use remained low in both groups (3.5% vs. 2.5%).

In terms of specialist clinical involvement, those with CKD were more likely to have contact with nephrology (79.8% vs. 20.2%) and cardiology (59.3% vs. 40.7%), whereas endocrinology input was more common in participants without CKD (63.3%). Baseline demographic and clinical characteristics are summarised in Table 1.

**TABLE 1:** Demographic and clinical profile of individuals with T1D, stratified by CKD status based on eGFR <60 mL/min.

Characteristic	Overall (*n* = 1106)	With CKD (*n* = 426)	Without CKD (*n* = 680)	Missing (%)
Age, mean (±SD)	44.1 (±17.7)	56.7 (±16.3)	36.1 (±13.4)	—
Age, *n* (%)				
18–28	272 (24.6)	25 (5.9)	247 (36.3)	
29–41	257 (23.2)	49 (11.0)	208 (30.6)	
42–54	289 (26.1)	125 (29.3)	164 (24.1)	
≥55	288 (26.0)	227 (53.3)	61 (9.0)	
Sex, *n* (%)				—
Female	459 (41.5)	194 (45.5)	265 (39.0)	—
Male	647 (58.5)	232 (54.5)	415 (61.0)	—
Ethnicity, *n* (%)				—
White	577 (52.2)	220 (51.6)	357 (52.5)	—
Mixed	355 (32.1)	137 (32.2)	218 (32.1)	—
Ethnic Minority background	64 (5.8)	12 (2.8)	52 (7.7)	—
Missing	110 (10.0)	57 (13.4)	53 (7.8)	—
Index of Multiple Deprivation (IMD), *n* (%)				—
1 (least deprived)	71 (6.4)	31 (7.3)	40 (5.9)	
2	93 (8.4)	36 (8. 5)	57 (8.4)	
3	142 (12.9)	55 (12.9)	87 (12.8)	
4	255 (23.1)	88 (20.7)	167 (24.6)	
5 (most deprived)	543 (49.2)	216 (50.7)	327 (48.2)	0.2
Smoking status, *n* (%)				—
Non‐smoker	396 (35.8)	136 (31.9)	260 (38.2)	
Ex‐smoker	376 (34.0)	186 (43.7)	190 (27.9)	
Smoker	334 (30.2)	104 (24.4)	230 (33.8)	
BMI, mean (±SD)	27.5 (±9.2)	28.3 (±12.2)	27.0 (±6.8)	3.1
Mean baseline eGFR, mL/min/1.73 m^2^ (±SD)	67.7 (±33.2)	33.5 (±18.0)	89.1 (±20.0)	—
Mean baseline uACR, mg/mmol (±SD)	8.4 (±37.9)	15.9 (±51.2)	0.5 (±1.2)	6.3
HbA1c, mmol/mol (±SD)	69.4 (±23.0)	68.9 (±22.7)	69.6 (±23.2)	—
Medications, *n* (%)				—
ACE inhibitor or ARB	585 (52.9)	312 (73.2)	273 (40.2)	
GLP‐1 receptor agonist	32 (2.9)	15 (3.5)	17 (2.5)	
SGLT2 inhibitor	143 (13.0)	66 (15. 5)	77 (11.3)	
Total clinical encounters in the observation period, *n* (%)				—
Endocrinology	855 (77.3)	314 (36.7)	541 (63.3)	
Nephrology	119 (10.8)	95 (79.8)	24 (20.1)	
Cardiology	258 (23.3)	153 (59.3)	105 (40.7)	

### 3.1 CKD onset and associated factors over the follow‐up period

The mean follow‐up period was 7.3 (±4.5) years, with a total person‐time at risk of 5203 days (see Table S1). A total of 426 CKD onset events occurred during follow‐up, with incidence rates increasing markedly with age—from 12.0 per 1000 person‐years (95% CI: 8.0–17.9) in those aged 18–28 years to 127.7 (111.4–146.3) in participants aged 55 years and older. Females had a higher incidence rate than males (53.4 vs. 42.6 per 1000 person‐years), and ex‐smokers showed the highest incidence among smoking groups (65.4; 56.2–76.1). Rates were broadly similar across deprivation quintiles and ethnic groups, except for a higher rate among participants with missing ethnicity data (79.5; 60.7–104.0). Those using ACE inhibitors or ARBs had a higher incidence (63.8; 56.7–71.8) compared with non‐users (27.5; 22.7–33.3), while rates did not differ substantially by use of SGLT2 inhibitors or GLP‐1 receptor agonists.

The mean follow‐up period for CKD defined by albuminuria (uACR ≥3 mg/mmol) was 5.1 (±4.6) years, with a total person‐time at risk of 5124 days (see Table S2). During this period, 475 participants developed CKD. Incidence rates increased with age, rising from 34.1 per 1000 person‐years (95% CI: 24.4–47.8) among those aged 18–28 years to 100.4 (85.6–117.9) in participants aged 55 years and older. Females exhibited a higher incidence than males (70.8 vs. 57.5 per 1000 person‐years). Incidence rates were broadly similar across ethnic groups, although higher among participants with missing ethnicity data (105.9; 78.8–142.4). Rates varied by deprivation, with the highest observed in quintiles 2 and 5 (71.1 and 71.4, respectively). Current and ex‐smokers had similarly elevated rates (around 70 per 1000 person‐years), compared with those with unknown smoking status (50.7). Use of statins and ACE inhibitors/ARBs was associated with higher incidence (83.9) than non‐use (35.4). Participants on SGLT2 inhibitors and GLP‐1 receptor agonists also showed higher incidence rates (80.6 and 104.8, respectively) compared with non‐users.

### 3.2 Factors associated with CKD by eGFR in individuals with T1D

**FIGURE 2:**
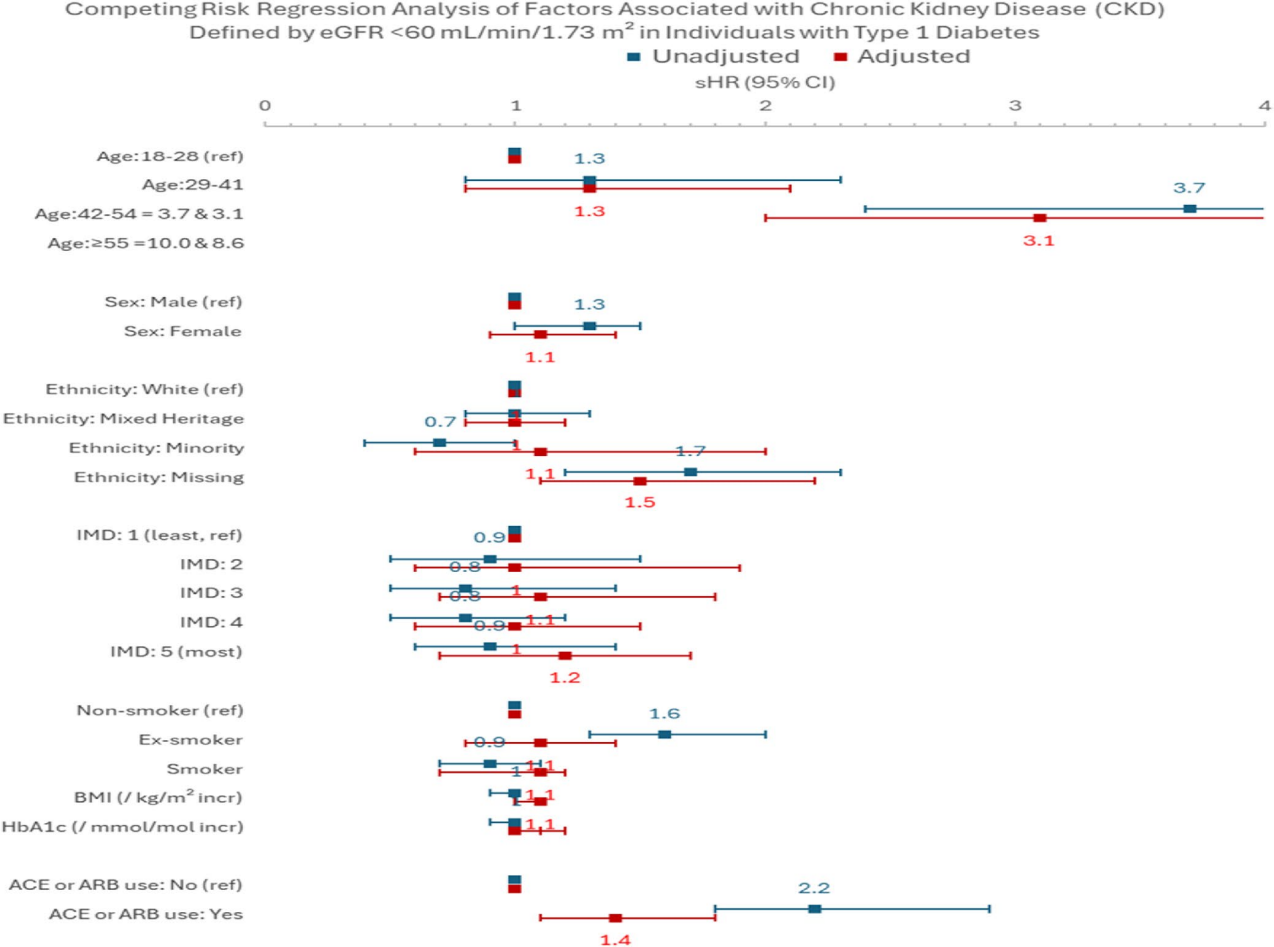
Competing risk regression analysis of factors associated with chronic kidney disease (CKD) defined by eGFR <60 mL/min/1.73 m^2^ in individuals with T1D.

In the competing risk regression analysis (see Figure 2), CKD was defined as an eGFR <60 mL/min/1.73 m^2^.

Increasing age was strongly associated with a higher risk of CKD in both unadjusted and adjusted models. Compared with participants aged 18–28 years, those aged 42–54 years had an adjusted sHR of 3.1 (95% CI: 2.0–5.0), while those aged 55 years and above had an sHR of 8.6 (95% CI: 5.4–13.9). Female sex was linked with a slightly higher risk of CKD in the unadjusted analysis (sHR 1.3; 95% CI: 1.0–1.5), although this association attenuated after adjustment (sHR 1.1; 95% CI: 0.9–1.4). Ethnicity was not significantly associated with CKD risk after adjustment; however, missing ethnicity data itself was associated with a higher risk (adjusted sHR 1.5; 95% CI: 1.1–2.2). No clear gradient in CKD risk was observed across deprivation quintiles. Ex‐smoking status was initially associated with an increased risk of CKD in unadjusted analyses (sHR 1.6; 95% CI: 1.3–2.0), but this association was no longer significant after adjustment (sHR 1.1; 95% CI: 0.8–1.4), and current smoking showed no significant association with CKD risk. Higher BMI was modestly associated with increased CKD risk in the adjusted model (sHR 1.1; 95% CI: 1.0–1.1). Lastly, participants receiving ACE inhibitors or ARBs had a higher risk of CKD compared to those who did not (adjusted sHR 1.4; 95% CI: 1.1–1.8).

### 3.3 Factors associated with CKD defined by albuminuria in individuals with T1D

**FIGURE 3:**
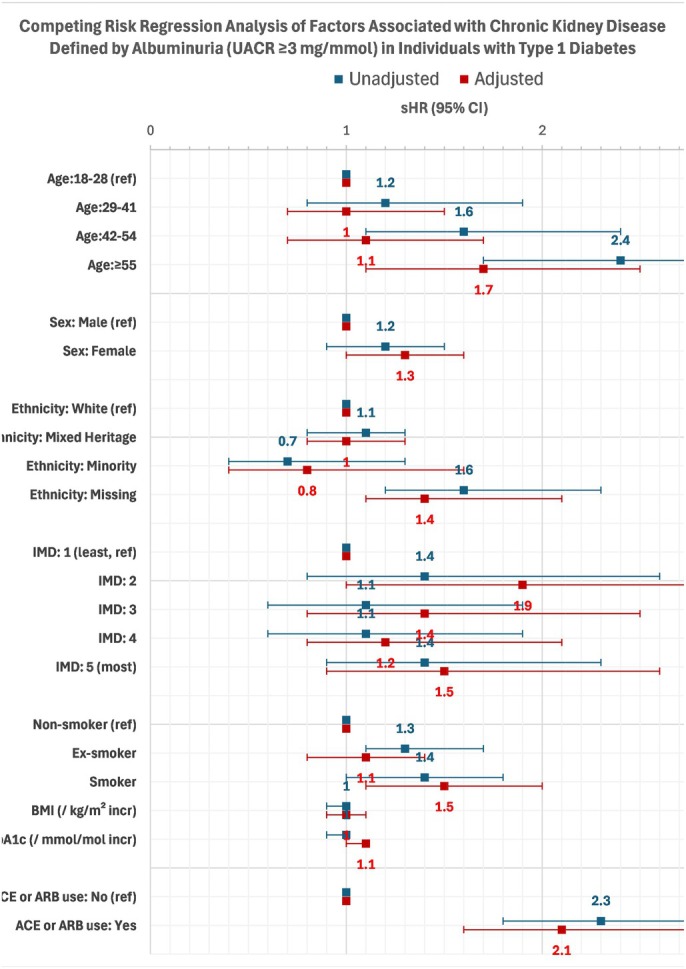
Competing risk regression analysis of factors associated with chronic kidney disease defined by albuminuria (UACR ≥3 mg/mmol) in individual with T1D.

Albuminuria data were available for 1006 individuals with T1D. Of these, 47.2% had a uACR of ≥3 mg/mmol. Figure 3 presents the results of the competing risk regression analysis examining factors associated with CKD defined by albuminuria.

Older age was associated with a higher risk of albuminuria, although this association was attenuated after adjustment. Compared with participants aged 18–28 years, those aged ≥55 years had an adjusted sHR of 1.7 (95% CI: 1.1–2.5), whereas the 42–54 years age group did not show a statistically significant increase in risk (sHR 1.1; 95% CI: 0.7–1.7).

After adjustment, female sex was significantly associated with an increased risk of albuminuria (sHR 1.3; 95% CI: 1.0–1.6). Ethnicity itself was not significantly associated, although participants with missing ethnicity data had a higher risk (sHR 1.4; 95% CI: 1.1–2.1). Socioeconomic deprivation showed some evidence of association: individuals in the second IMD quintile had an adjusted sHR of 1.9 (95% CI: 1.0–3.5) compared with the least deprived, while other quintiles did not show significant associations.

Smoking status was also important: current smokers had a significantly higher risk of albuminuria compared with non‐smokers (adjusted sHR 1.5; 95% CI: 1.1–2.0). Ex‐smokers, however, did not differ significantly from non‐smokers (sHR 1.1; 95% CI: 0.8–1.4). Higher HbA1c levels were independently associated with increased risk, with each mmol/mol rise corresponding to an sHR of 1.1 (95% CI: 1.0–1.1).

Finally, the use of ACE inhibitors or ARBs was associated with a higher prevalence of CKD in unadjusted models (sHR 2.3; 95% CI: 1.8–3.0), and this association remained significant after adjustment for demographic and clinical factors (adjusted sHR 2.1; 95% CI: 1.6–2.9).

## 4 DISCUSSION

In this cohort study of adults with T1D, we observed a substantial burden of CKD, with 38.5% of participants showing reduced kidney function (eGFR <60 mL/min/1.73 m^2^) and 47.2% exhibiting significant albuminuria (≥3 mg/mmol) over the follow‐up period. Notably, 23.8% met both criteria, indicating more advanced kidney involvement. These findings underscore the persistent and considerable burden of CKD in this population, which continues to pose a significant clinical challenge.

Compared with other published studies, our observed prevalence is higher. Tuttle et al.^5^ reported a CKD prevalence of 27.1% among adults with T1D in a large US cohort, whereas Rossing et al.^4^ reported 25.0%. Kristófi et al.^8^ found prevalence estimates of around 18% in a two‐country Scandinavian study, and Swartling et al.^9^ reported similarly lower figures in Sweden. The single‐centre study by Majeed et al.^10^ also documented a lower prevalence than observed here. The higher cumulative incidence in our study may reflect differences in study design and population characteristics, including age, as well as persisting health inequalities; nearly half of our cohort resides in the most deprived areas, which may affect access to reno‐protective therapies and risk factor management. Collectively, these findings highlight the need for earlier detection, targeted interventions and equitable care models.

Age emerged as the strongest independent predictor of CKD across both eGFR and albuminuria definitions, reflecting the cumulative impact of diabetes duration, vascular ageing and co‐morbidities on renal decline. This aligns with large cohort studies.^5^, ^9^ Higher BMI was also consistently associated with increased CKD risk, supporting calls to prioritise weight management and metabolic health in CKD prevention. Baseline HbA1c did not differ significantly between participants with and without CKD; however, after adjustment, higher HbA1c was independently associated with increased risk of albuminuria and, to a lesser extent, reduced eGFR, emphasising the importance of optimising glycaemic control over time.

In our cohort, smoking was linked to higher albuminuria, whereas ex‐smoking showed no significant association, highlighting the benefits of cessation. A higher proportion of participants with CKD were ex‐smokers, suggesting quitting is achievable following diagnosis or complication onset. Smoking remains a key modifiable risk factor in T1D, with prevalence ranging from 10% to 30% across studies and up to 39% internationally.^11^ These findings underscore the need for targeted interventions, including counselling, pharmacotherapy and ongoing follow‐up, to reduce cardiovascular and kidney disease risk.

Reno‐protective medications, including ACE inhibitors and ARBs, were commonly prescribed, reflecting current guidelines. The higher prevalence of CKD among those receiving these treatments may indicate suboptimal dosing or discontinuation, as noted by Tuttle.^5^ Despite these therapies, many individuals with T1D continue to develop CKD, highlighting the need to investigate newer agents such as SGLT2 inhibitors and GLP‐1 receptor agonists.

In this cohort, SGLT2 inhibitor use was higher in the CKD group (15.5% vs. 11.3%), while GLP‐1 receptor agonist use remained minimal (3.5% vs. 2.5%). This reflects limited licensing at the time, although dapagliflozin had an indication for T1D prior to licence withdrawal. Emerging evidence suggests these agents may offer renal and metabolic benefits: Shah et al.^12^ showed semaglutide improved time in range, HbA1c, and weight in adults with T1D and obesity without increasing severe hypoglycaemia or ketoacidosis, and Sridhar et al.^13^ demonstrated similar benefits of sotagliflozin in T1D patients with and without CKD.

Our analysis did not find a significant association between ethnicity and CKD onset, contrasting with studies such as Mangelis et al.,^14^ which identified African Caribbean ethnicity as a predictor of rapid kidney function decline. The discrepancy may reflect the low proportion of non‐Caucasian participants and missing ethnicity data in our cohort. Consistent with emerging evidence, however, earlier CKD onset in ethnic minority populations highlights the need for targeted interventions and monitoring.^15^, ^16^, ^17^

Finally, endocrinology consultations were more common among participants without CKD, whereas nephrology and cardiology visits predominated in those with CKD, reflecting the interconnected burden of diabetes, kidney disease and cardiovascular complications.^18^ This emphasises the patient burden and the importance of integrated, multidisciplinary care. The model of care described in our previous paper offers a framework to bridge clinical and policy gaps by promoting coordinated management across specialties.^19^ Adopting such approaches may reduce fragmentation, ease patient burden and ultimately improve outcomes for those living with multiple chronic conditions, taking into account the link between incident diabetes, kidney disease and associated co‐morbid conditions.^20^

### 4.1 Strengths and limitations

This study benefits from a large, well‐characterised cohort of individuals with T1D and utilises routinely collected clinical data, enabling a pragmatic, real‐world evaluation of factors associated with CKD. To our knowledge, this is the first large‐scale cohort analysis of T1D and CKD in the UK. The use of competing risk regression models is a key strength, appropriately accounting for death as a competing event. Additionally, the inclusion of relevant covariates such as age, sex, ethnicity, deprivation, smoking status, BMI and HbA1c strengthens the analytical approach.

However, there are notable limitations. The study did not adjust for the duration of diabetes, a key determinant of CKD risk, nor for blood pressure or cholesterol levels, both of which are known to influence albuminuria and the decline in eGFR. Furthermore, we lacked retinopathy data for the cohort, which is a strong indicator of microvascular disease. Additionally, it is possible that some of the patients included in the analysis may have type 3C diabetes, as misclassification and incorrect coding are well‐known issues in primary care datasets. This may have introduced residual confounding. Finally, the observational nature of the study precludes causal inference.

## 5 CONCLUSION

In this large, real‐world cohort of adults with T1D, we observed a substantial burden of CKD, driven by both non‐modifiable factors such as age and modifiable risks including higher BMI, suboptimal glycaemic control and smoking. Despite widespread use of ACE inhibitors and ARBs in line with current guidance, uptake of newer reno‐protective therapies, such as SGLT2 inhibitors and GLP‐1 receptor agonists, remain limited, reflecting gaps in routine care. These findings underline the importance of earlier detection, proactive risk factor management and broader adoption of emerging treatments to address the continuing challenge of CKD in this population and improve long‐term health outcomes.

## FUNDING INFORMATION

The authors received no financial support for the research, authorship and/or publication of this article.

## CONFLICT OF INTEREST STATEMENT

HHH‐A received speaker honoraria from AstraZeneca, NovoNordisk and Bayer. MS, AF and AH have declared no conflicts of interest. DCW has an ongoing consultancy contract with AstraZeneca and has received payments for consultancy and/or speaking activities from multiple companies, including Amgen, Astellas, Bayer, Boehringer Ingelheim, Eledon, GSK, Galderma, Gilead, Janssen, Mundipharma, Menarini, MSD, NovoNordisk, Pharmacosmos, Tricida and Vifor. JK has received honoraria for delivering educational meetings and/or attending advisory boards from Boehringer Ingelheim, AstraZeneca, Sanofi, NAPP and research grants from AstraZeneca and Sanofi.

## Supporting information


**Table S1.** CKD onset defined as eGFR <60 mL/min: rates by participant characteristics (per 1000 person‐years).
**Table S2.** CKD defined by albuminuria (≥3 mg/mmol): rates by participant characteristics (per 1000 person‐years).
