# Supplementary material for: Incidence and risk factors for chronic kidney disease in individuals with type 1 diabetes: A population‐based study in Salford, Manchester
Source: Diabet Med. 2025 Nov 20;43(1):e70175. doi: 10.1111/dme.70175 (PMC12700510; doi:10.1111/dme.70175)
Supplement: Supplementary file 1 — Table S1. CKD onset defined as eGFR <60 mL/min: rates by participant characteristics (per 1000 person‐years). Table S2. CKD defined by albuminuria (≥3 mg/mmol): rates by participant characteristics (per 1000 person‐years). [file DME-43-e70175-s001.docx]

Supplemental File 1 – Table 1. CKD onset defined as eGFR < 60 ml/min: Rates by Participant Characteristics (per 1000 person-years)

| Characteristic | Category | Events (D) | Person-years (Y) | Rate | 95% CI |
| --- | --- | --- | --- | --- | --- |
| Age group (years) | 18–28 | 24 | 2.00 | 12.0 | (8.0–17.9) |
|  | 29–41 | 35 | 2.11 | 16.6 | (11.9–23.1) |
|  | 42–54 | 114 | 2.38 | 47.8 | (39.8–57.4) |
|  | ≥55 | 208 | 1.63 | 127.7 | (111.4–146.3) |
| Sex | Male | 209 | 4.90 | 42.6 | (37.2–48.8) |
|  | Female | 172 | 3.22 | 53.4 | (46.0–62.0) |
| Ethnicity | White | 194 | 4.39 | 44.2 | (38.4–50.8) |
|  | Mixed | 123 | 2.71 | 45.4 | (38.1–54.2) |
|  | Missing | 53 | 0.67 | 79.5 | (60.7–104.0) |
|  | Ethnic minority | 11 | 0.36 | 30.6 | (16.9–55.3) |
| Deprivation (IMD) | 1 (most deprived) | 25 | 0.50 | 50.3 | (34.0–74.5) |
|  | 2 | 34 | 0.73 | 46.9 | (33.5–65.6) |
|  | 3 | 51 | 1.15 | 44.2 | (33.6–58.2) |
|  | 4 | 79 | 1.92 | 41.2 | (33.0–51.3) |
|  | 5 (least deprived) | 192 | 3.82 | 50.3 | (43.7–57.9) |
| Smoking status | Unknown | 121 | 2.96 | 40.8 | (34.2–48.8) |
|  | Ex‑smoker | 168 | 2.57 | 65.4 | (56.2–76.1) |
|  | Current smoker | 92 | 2.60 | 35.4 | (28.9–43.5) |
| ACEi/ARB use | No | 104 | 3.78 | 27.5 | (22.7–33.3) |
|  | Yes | 277 | 4.34 | 63.8 | (56.7–71.8) |
| SGLT2 inhibitor | No | 327 | 7.02 | 46.6 | (41.8–51.9) |
|  | Yes | 54 | 1.11 | 48.9 | (37.4–63.8) |
| GLP‑1 use | No | 368 | 7.87 | 46.8 | (42.2–51.8) |
|  | Yes | 13 | 0.26 | 50.8 | (29.5–87.5) |

Supplemental File 1 – Table 2. CKD defined by albuminuria (≥ 3 mg/mmol): Rates by Participant Characteristics (per 1000 person-years)

| Variable | Characteristic | D | Person-years | Rate | 95% CI |
| --- | --- | --- | --- | --- | --- |
| Age | 18–28 | 34 | 0.9958 | 34.1 | 24.4–47.8 |
|  | 29–42 | 55 | 1.2555 | 43.8 | 33.6–57.1 |
|  | 43–54 | 85 | 1.4044 | 60.5 | 48.9–74.9 |
|  | ≥55 | 150 | 1.4934 | 100.4 | 85.6–117.9 |
| Sex | Male | 176 | 3.0584 | 57.5 | 49.6–66.7 |
|  | Female | 148 | 2.0907 | 70.8 | 60.3–83.2 |
| Ethnicity | White | 166 | 2.8426 | 58.4 | 50.2–68.0 |
|  | Mixed | 105 | 1.6886 | 62.2 | 51.4–75.3 |
|  | Ethnic minority | 9 | 0.2026 | 44.4 | 23.1–85.4 |
|  | Missing ethnicity | 44 | 0.4153 | 105.9 | 78.8–142.4 |
| IMD Quintile | 1 | 18 | 0.3767 | 47.8 | 30.1–75.8 |
|  | 2 | 31 | 0.4360 | 71.1 | 50.0–101.1 |
|  | 3 | 40 | 0.7402 | 54.0 | 39.6–73.7 |
|  | 4 | 64 | 1.1957 | 53.5 | 41.9–68.4 |
|  | 5 | 171 | 2.3943 | 71.4 | 61.5–83.0 |
| Smoking status | Unknown smoking | 98 | 1.9318 | 50.7 | 41.6–61.8 |
|  | Ex-smoker | 127 | 1.8193 | 69.8 | 58.7–83.1 |
|  | Current smoker | 99 | 1.3980 | 70.8 | 58.2–86.2 |
| Statins | Not on statins | 79 | 2.2298 | 35.4 | 28.4–44.2 |
|  | On statins | 245 | 2.9193 | 83.9 | 74.0–95.1 |
| ACR or ARB | Not on ACR/ARB | 79 | 2.2298 | 35.4 | 28.4–44.2 |
|  | On ACR/ARB | 245 | 2.9193 | 83.9 | 74.0–95.1 |
| SGLT2 inhibitor | Not on SGLT2 | 273 | 4.5165 | 60.4 | 53.7–68.1 |
|  | On SGLT2 | 51 | 0.6326 | 80.6 | 61.3–106.1 |
| GLP-1 receptor agonist | Not on GLP1 | 308 | 4.9964 | 61.6 | 55.1–68.9 |
|  | On GLP1 | 16 | 0.1527 | 104.8 | 64.2–171.0 |
